# Patent Foramen Ovale Closure in Special Clinical Situations: More Questions Than Answers?

**DOI:** 10.3390/life14060706

**Published:** 2024-05-30

**Authors:** Anastasios Apostolos, Polyxeni Alexiou, Amalia Papanikolaou, Georgios Trantalis, Maria Drakopoulou, Nikolaos Ktenopoulos, Ioannis Kachrimanidis, Panayotis K. Vlachakis, Ismini Tsakiri, Grigorios Chrysostomidis, Konstantina Aggeli, Costas Tsioufis, Konstantinos Toutouzas

**Affiliations:** 1 First Department of Cardiology, Medical School, National and Kapodistrian University of Athens, Hippocration General Hospital, 115 27 Athens, Greece; anastasisapostolos@gmail.com (A.A.); alexioupolyxeni@gmail.com (P.A.); geotrantalis@gmail.com (G.T.); mdrakopoulou@hotmail.com (M.D.); nikosktenop@gmail.com (N.K.); iskachrimanidis@gmail.com (I.K.); vlachakispanag@gmail.com (P.K.V.); ismini.tsakiri1@gmail.com (I.T.); dina.aggeli@gmail.com (K.A.); ktsioufis@gmail.com (C.T.); 2 Department of Cardiology and Angiology, Universitatklinikum Essen, 451 47 Essen, Germany; amaliapap22@gmail.com; 3 Second Department of Adult Cardiac Surgery, Onassis Cardiac Surgery Center, 176 74 Athens, Greece; gregory.chrisostomidis@gmail.com

**Keywords:** patent foramen ovale, PFO, decompression illness, migraine, thrombophilia, older patients

## Abstract

Patent foramen ovale (PFO) is a remnant of the foetal circulation resulting from incomplete occlusion of the septum primum and septum secundum. Although prevalent in about 25% of the population, it mainly remains asymptomatic. However, its clinical significance in situations such as cryptogenic stroke, migraine, and decompression illness (DCI) has been well described. Recent randomised clinical trials (RCTs) have demonstrated the efficacy of percutaneous PFO closure over pharmacological therapy alone for secondary stroke prevention in carefully selected patients. Notably, these trials have excluded older patients or those with concurrent thrombophilia. Furthermore, the role of closure in other clinical conditions associated with PFO, like decompression sickness (DCS) and migraines, remains under investigation. Our review aims to summarise the existing literature regarding epidemiology, pathophysiological mechanisms, optimal management, and closure indications for these special patient groups.

## 1. Introduction

Patent foramen ovale (PFO) is the most prevalent heart interatrial communication, affecting about 25% of the global population, which equates to around 1.9 billion people worldwide [1]. PFO is mandatory during foetal life because it helps the delivery of oxygenated blood by connecting atria and bypassing lungs. After birth, blood oxygenation is performed by the lungs; thus, PFO does not serve any purpose in human physiology and typically closes during development. Nevertheless, some individuals retain a PFO independently or in conjunction with other related heart conditions, such as atrial septal aneurysms, Eustachian valves, Chiari networks, and Ebstein’s anomaly [2,3,4].

A causal relationship between PFO and cryptogenic stroke through paradoxical thromboembolism has been established, with PFO-associated stroke representing about 10% of ischemic strokes in patients under 60 years old [5,6,7]. Moreover, PFO has also been linked to the pathogenesis of conditions such as decompression illness (DCI) and migraine, among others. Nonetheless, the precise association and causality are yet to be proven and are sometimes questioned, especially when considering the high prevalence of PFO [8].

The first attempts of PFO closure for secondary stroke prevention date back more than 30 years [9]. Nowadays, percutaneous PFO closure is considered a safe procedure, usually performed under transoesophageal echocardiography (TEE) guidance [10]. Various devices have been used, with the Amplatzer PFO Occluder and the Gore Cardioform Septal Occluder being the only two FDA-approved options.

Large-scale studies have demonstrated 96% successful implantations, with an equivalent rate of complete closure after one year, as well as low peri- and postoperative complications [10,11]. Although neither major nor common, short- or long-term complications like atrial fibrillation (AF) do exist and should be recognised [12,13,14,15].

To date, evidence based on randomised clinical trials (RCTs) strongly supports percutaneous PFO closure over solely antithrombotic treatment for secondary prevention in selected patients ≤ 60 years old diagnosed with cryptogenic ischemic stroke [16,17,18,19]. Based on these trials, both European and American guidelines recommend percutaneous PFO closure over antiplatelet therapy alone for patients aged 18–60/65 years who have a prior PFO-associated stroke, supported by clinical, anatomical, and imaging justification. This recommendation is strongly endorsed [11,20]. However, older patients with cryptogenic stroke or known thrombophilia were excluded from such RCTs. Meanwhile, the beneficial role of PFO closure in other diseases linked with PFO, such as DCI, migraines, or platypnoea-orthodeoxia syndrome, remains questionable.

The purpose of our review is to summarise the current knowledge regarding epidemiology, pathophysiology, and the role of closure on these patients to help clinicians in the decision-making process and tailored approach.

## 2. Decompression Illness

Self-contained underwater breathing apparatus (SCUBA) diving, although generally considered safe, can result in several mild or more threatening complications, including cephalalgia, sinus disorders, and DCI, with an overall rate of 3.02 per 100 dives. Self-documented decompression sickness (DCS) occurred at a rate of 1.55 per 10^3^ dives in a study, while medically treated DCS approached about 6 per 10^5^ dives.

The cause of DCI, either in the form of arterial gas embolism (AGE) or DCS, is primarily the rapid radical decrease in environmental pressure (decompression) during the diver’s ascent that leads to intra- or extravascular bubbles formation [21]. In AGE, embolic venous or alveolar gas bubbles infiltrate into the arterial circulation via a right-to-left shunt (RLS) or pulmonary vessels with possible ischemic complications. In DCS, which is more frequent, bubble development or enlargement occurs in situ from a dissolved inert gas, mainly nitrogen, based on the principle that the higher the partial gas pressure is, the more gas dissolves in tissues. As the diver ascents, the ambient pressure may fall rapidly compared to the dissolved gas pressure in tissues, leading to hypersaturation [22,23]. DCI affects not only divers, but also pilots, air workers, and astronauts, and may also be iatrogenic in the case of AGE [24].

DCI is subdivided into two types: type I, referring to minor, non-systematic manifestations such as pain, cutaneous or constitutional symptoms, and type II, with more serious complications, including weakness, paraesthesias, paralysis and neurological signs, cardiac arrest, or even death [24]. While venous gas emboli are common after diving, they are typically filtered by the lungs, remaining asymptomatic. However, if they exceed pulmonary capacity and a PFO is present, some of these emboli could enter the arterial circulation, potentially leading to disease [22]. Divers with PFO are at higher risk of developing DCS. The actual incidence remains under investigation, and it varies between 2.5 to 6 times higher than that of individuals without a PFO [8,25,26,27,28].

Early onset DCS, even during ascent or immediately after straining; type II neurological symptoms; as well as the diagnosis of cutis marmorata, and diving within safe limits of no-decompression, indicate a higher possibility of PFO presence [8,29,30]. In professional divers who suffered from PFO-associated DCS, the size of the PFO is a predictor of recurrence. Additionally, Honěk et al. analysed a total of 169,411 dives and showed that a large PFO was the only major risk factor for unprovoked DCS compared to controls [31]. Torti et al. also concluded that the risk of major DCI is strongly correlated with PFO size [27].

After the manifestation of a probable DCS, a comprehensive medical evaluation is crucial to identify the contributing risk factors. Diagnosis is mainly clinical and includes risk stratification based on diving practices, anatomical features, and functional studies. Following the latest European recommendations, PFO testing should be considered when no clear risk factors for DCS are present or when a high but non-modifiable risk for developing DCS is expected.

Primary routine screening for PFO in divers is not recommended. Screening is advised only for high-risk divers, such as those with a recurrent DCI event, as well as cryptogenic stroke, migraine with aura (MA+), congenital heart disease, or a family history of PFO/atrial septal defect [8,20,30]. However, a recent study supports screening even in low-risk patients for DCS; they followed up consecutive divers from their registry who continued diving and concluded that screening and risk stratification significantly decreased the DCS occurrence among divers with PFO compared to controls [32].

In the presence of a PFO in a diver, the available options are synopsised as follows: (1) diving cessation, (2) adoption of conservative strategies, and (3) percutaneous PFO closure [30].

The first percutaneous PFO closure to avert DCI recurrence was performed back in 1996 [33]. Most available studies depict an advantage of PFO closure in reducing DCI recurrence incidence (Table 1). However, these data are mainly observational, quite heterogeneous, with a limited number of participants, and sometimes with incomplete follow-up. Noteworthy, PFO closure does not protect from other causes of DCS, such as pulmonary barotrauma, as a PFO is not the sole DCS cause. Given the low overall incidence of DCI events, which is further decreased when conservating diving techniques are utilised and recognising that PFO closure is not an entirely risk-free procedure, the question of whether the procedure should be routinely performed for divers with a previous DCI event or, generally, a PFO, remains controversial.

European guidelines recommend conservative methods as the first-line treatment after a DCS, regardless of PFO presence. In cases where (a) a high predicted causal relationship between DCS and PFO exists, (b) activity discontinuation is not an option, (c) conservative methods are not applicable or effective, and d) the risk of DCS recurrence even after behavioural changes is still considered high, PFO closure could be considered. Noteworthy, they highlight the importance of ensuring total PFO closure after the intervention, before the patient returns to diving practice [8]. SCAI guidelines also align with that perspective, suggesting against PFO closure for all divers with prior DCI. They propose that it can be individually recommended to patients for whom the possible benefit of closure outweighs the potential harm and complication risks [20]

## 3. Migraine

Migraine stands as one of the most prevalent disabling conditions worldwide, affecting approximately 12–15% of the population [41,42]. Aura, whether occurring before, during, or without a headache, manifests in 25–30% of migraineurs [43,44]. The presence of aura has not been associated with particular risk factors (e.g., younger patients, women) or comorbidities, including PFO. To the best of our knowledge, Del et al., in 1998, were the first to report a higher prevalence of RLS in patients with MA+ compared to controls [45]. Subsequent studies have suggested a causal relationship between the presence of PFO and MA+, which eventually raised the question of whether PFO closure could serve as a cure for migraine attacks. The prevalence of PFO in migraine patients has shown wide variation across observational studies, ranging from 26.8–96% in MA+ compared to 16.2–44.4% in Migraine without aura (MA-) patients [46,47,48,49,50,51,52,53].

A variety of mechanisms have been proposed linking PFO and migraines. According to one theory, PFO allows metabolic compounds or micrο-emboli, normally filtered by the lungs, to enter the arterial circulation. If these accumulate to sufficient levels and cross the blood–brain barrier, that can result in vascular network and trigmental nerve irritation, subsequently leading to migraine [54,55]. Similarly, 5-hydroxytryptamine (5-HT) is normally metabolised in the lungs by monoamine oxidase. In the case of a PFO, the 5-HT blood rise in the blood that enables platelet activation and aggregation is thought to act as a possible migraine stimulus [56]. A recent article by Trabattoni et al. found that the presence of a PFO maintains a prothrombotic state possibly due to oxidative stress that may also be related to 5-HT dysregulation [57]. Another hypothesis is that because a PFO induces arterial oxygen desaturation and therefore plasminogen activator-1 expression that inhibits fibrinolysis, the possibility of a paradoxical embolism rises. That triggers cortical spreading depression which is considered a central factor of a migraine attack [54,58]. Additionally, the higher occurrence of PFO in migraineurs implies a possible genetic predisposition [59].

Several studies have explored the role of PFO closure in patients with migraines. Their main characteristics are synopsised in Table 2. Three RCTs have also been completed, the Migraine Intervention with STARFlex Technology (MIST) [60], the Percutaneous Closure of PFO in Migraine with Aura (PRIMA) [61], and the Prospective, Randomized Investigation to Evaluate Incidence of Headache Reduction in Subjects with Migraine and PFO Using the AMPLATZER PFO Occluder to Medical Management (PREMIUM) trial [62]. Contrary to contemporary observational studies, the RCTs failed to reach their primary endpoints and demonstrated a statistically significant role of PFO/RLS closure in migraine cessation.

A recent pooled analysis of PREMIUM and PRIMA trials by Mojadidi et al. revealed that PFO closure was a safe procedure with a statistically significant decrease in the number of migraine days (3.1 vs. 1.9; *p* = 0.02) and attacks (2.0 vs. 1.4; *p* = 0.01) per month, as well as in the complete migraine cessation rate (9% vs. 0.7%; *p* < 0.001) compared to the control group. They suggested that individuals with frequent aura derived the greatest benefit, possibly due to a different underlying pathophysiological mechanism [69]. The meta-analysis of the European position paper, encompassing the three RCTs and 22 observational studies, revealed a statistically significant benefit of PFO closure compared to medical treatment for MA+. However, the authors emphasised that the level of evidence supporting this conclusion was considerably low and primarily derived from positive observational studies rather than the RCTs. Consequently, the advantage remains on a hypothetical level for implementation to all migraineurs [8]. An earlier meta-analysis of Butera et al., incorporating data from the MIST trial and observational studies, focused on migraineurs with a history of a cerebrovascular accident (CVA). The results indicated that when treated for this condition, they also experienced migraine relief [70].

There are currently three ongoing studies with substantial patient populations evaluating the safety and efficacy of PFO closure in migraineurs. These include two RCTs with a sham procedure control group and a randomised case-control study. In the RCT GORE^®^ CARDIOFORM Septal Occluder Migraine Clinical Study (RELIEF) (clinicaltrials.gov: NCT04100135), patients will also receive thienopyridines, and in the COMParison of the EffecT of dEvice Closure in Alleviating Migraine with PFO (COMPETE-2) phase four double-blind RCT (clinicaltrials.gov: NCT05561660), the control group will receive aspirin. The phase three multicenter case-control study, The Effectivity and Safety of PFO Closure vs Medicine in Alleviating Migraine (SPRING) study (clinicaltrials.gov: NCT04946734), will evaluate the addition of PFO closure to aspirin, clopidogrel, and triptans.

### Key Recommendations

Based on the available evidence, interventional therapy should not be considered an alternative to conservative methods for treating migraine. The European guidelines suggest that the initial steps in migraine management involve optimal medical treatment. If that proves ineffective, and only in cases where there is a concurrent CVA and/or in selected patients with severe, disabling MA+, PFO closure, if PFO presence is identified, can be considered off-label. This decision should be made after a multidisciplinary expert assessment and an evaluation of the individual’s needs [8]. The SCAI guidelines also align with the preceding statement for migraineurs without a history of a PFO-associated stroke [20].

## 4. Older Patients

While the association between PFO and cryptogenic stroke in patients under 60 years old has been established, evidence suggests that PFO also demonstrates a link with cryptogenic stroke in older patient groups [71]. The management of patients >60 years old with a cryptogenic stroke and a PFO remains controversial, as current RCTs excluded this age group. Handke et al. found the TEE-diagnosed prevalence of PFO in older patients with cryptogenic stroke to be 28.3% compared to 11.9% in the same age group with a defined stroke cause (Odds Ratio (OR) 2.92; 95% Confidence Interval (CI): 1.70-5.01, *p* < 0.001) [72]. Similarly, Mazzucco et al. reported a higher prevalence of RLS diagnosed with transcranial Doppler (TCD) in cryptogenic stroke patients >60 years compared to the same-aged patients with a known stroke cause (OR 2.06; 95% CI: 1.32-3.23, *p* = 0.001), indicating that the association persists in older patients. Extrapolating the findings to the British population, the results showed that 70.2% of cryptogenic stroke events associated with large PFO are predicted to affect even patients >60 years [73]. Based on data from four studies and a total of 2171 participants, the European guidelines showed an increased risk of recurrence in older patients with a previous PFO-associated stroke (Hazard Ratio (HR) 1.47, 95% CI 1.2–1.8) compared to younger individuals [11] Multiple reasons can explain this age-related increased risk. First, the possibility of venous thrombotic events is generally higher in older ages [74]. Pulmonary or right ventricular pressure pathologies have also been associated with the onset of PFO-related symptoms, which are more commonly met in older patients [75]. Lastly, other cardiovascular (CV) risk factors that are more prevalent in older individuals may contribute to the increased risk [71].

The DEFENSE-PFO was the only RCT with a small number of patients over 60 years of age. In the subgroup analysis, the difference in the 2-year event rate between the closure and the medical treatment group was statistically significant (24.6%; HR 7.36; 95% CI: 0.28–195.8; log-rank *p* = 0.07) [76]. However, when evaluating the power of its results, it is important to consider the small study size. The summary of the main characteristics of the observational studies is presented in Table 3.

At present, two observational studies and one RCT are ongoing on this topic. The CLOSE-2 trial (clinicaltrials.gov NCT05387954) is a phase three RCT aiming to assess the superiority of adding PFO closure to antiplatelet therapy in patients aged 60–80 years old after a PFO-associated stroke, compared to antiplatelet therapy alone. The study will also investigate the comparative efficacy of antiplatelet vs. anticoagulation therapy for preventing stroke recurrence. The COACH ESUS (clinicaltrials.gov NCT05238610) is a prospective observational study that is currently recruiting patients aged 60 and above with a high-risk PFO and Embolic Stroke of Undetermined Source (ESUS) to demonstrate the efficacy of PFO closure compared to medical treatment. The third ongoing study is the DefenseElderly (clinicaltrials.gov NCT04285918), an observational prospective cohort study. It aims to evaluate the clinical impact of AF in patients over 60 years old with ESUS and a high-risk PFO, comparing them to similar patients without high-risk PFO.

### Key Recommendations

The current guidelines for managing PFO lack definite recommendations for older patients with cryptogenic stroke. Decisions should be made in an interdisciplinary manner, taking into consideration the individual’s overall health. The consensus statement from the ESO-Karolinska Stroke Update Conference in 2019 concluded that for ages 60–65, PFO closure can be offered in conjunction with antiplatelet therapy as opposed to antiplatelet therapy alone. For individuals older than 65 years, the decision to substitute antiplatelet therapy with PFO closure is individualised [84]. According to the European guidelines, for patients older than 65 years of age, the choice between drug therapy and PFO closure depends on comorbidity factors and the risks associated with each method for every patient separately [11]. The American Academy of Neurology (AAN) recommends PFO closure for patients aged 60–65 with few CV risk factors, only when other potential stroke causes, including AF, have been ruled out [85]. SCAI guidelines suggest incorporating PFO closure into the treatment plan, in addition to antiplatelet therapy, for adults ≥60 with a PFO-associated stroke, after considering the patient’s preferences [20]. While current data may support considering PFO closure for adults over 60 years old, RCTs are warranted to confirm the effectiveness, safety, and potential superiority of the intervention compared to medical treatment alone.

## 5. Thrombophilia

Thrombophilia is a broad term that describes both inherited and acquired conditions that predispose individuals to a prothrombotic state [86]. Common hereditary types include Factor V Leiden (FVL), protein C and S or antithrombin deficiency, and prothrombin gene mutation (PTM). Antiphospholipid syndrome is the primary representative of acquired thrombophilia. Due to the diverse types, the incidence of thrombophilia varies widely, ranging from 0.1 to 20% in the general population. The long-known risk of venous thromboembolism is associated with inherited types, whereas acquired thrombophilia is linked to both venous and arterial thromboembolism episodes. Thrombophilia is commonly present among patients with PFO, with studies indicating a prevalence of 5–31% [87].

Studies on the connection between PFO-associated stroke and thrombophilia provided inconsistent results, with some not indicating an increased risk of stroke [88,89,90] or stroke recurrence in the presence of PFO [91]. Conversely, a meta-analysis by Pezzini et al. found a significant connection between PTM and PFO-associated stroke (OR 3.9, 95% CI 2.2–6.7) compared to non-PFO-associated stroke patients (2.3, 95% CI 1.2–4.4) [92]. Another study showed that FVL or PTM were associated with cryptogenic stroke (*p* = 0.022) compared to other hypercoagulable states (*p* = 0.140) [93]. Liu et al. concluded that in the presence of hypercoagulability, the risk for recurrent stroke/transient ischemic attack (TIA) was higher (HR: 1.85, 95% CI: 1.09–3.16; *p* = 0.024) [94]. A meta-analysis by Hviid et al. also showed that the presence of inherited or acquired thrombophilia significantly increased the risk of stroke recurrence after PFO-associated stroke (OR 2.41, 95% CI: 1.44–4.06) [95].

Current guidelines do not recommend thrombophilia screening before PFO closure. According to the AAN, thrombophilia screening can be considered in patients who will undergo PFO closure and for whom a hypercoagulable state, as a plausible high-risk stroke mechanism, it would alter the medical treatment management (e.g., young patients with APS) [85].

A study by Omran et al. assessed thrombophilia testing in 196 stroke patients, including 40 with a PFO. The results showed that only 16 patients (37% with PFO) had a management alteration. The presence of PFO was not associated with a positive thrombophilia test or modification in medical management. Consequently, the study concluded that universal thrombophilia testing is not justified [96]. The British Society of Hematology guidelines also state that there is insufficient evidence to support thrombophilia testing in patients with a PFO. Thus, routine thrombophilia testing in PFO-associated stroke is not indicated [86].

Although patients with thrombophilia were not included in the RCTs, evidence from observational studies shows a possible benefit of PFO closure (Table 4). A meta-analysis of 11 observational studies demonstrated a non-significant increase in the risk of TIA/stroke recurrence after PFO closure. The authors concluded that PFO closure could be considered at least a non-inferior approach, compared to antithrombotic therapy in patients with a PFO-associated stroke and thrombophilia [95,97].

### Key Recommendations

While existing data do not endorse PFO closure as the gold standard for patients with a previous PFO-associated stroke, it can be proposed as an alternative in patients with thrombophilia who do not receive anticoagulation. However, for patients under anticoagulants, evidence is still missing [95]. The European guidelines support PFO closure in hypercoagulable states in cases where there is a short-term requirement for anticoagulation or a high-risk recurrence probability despite the use of anticoagulation therapy [11]. The SCAI guidelines are in favour of routine PFO closure for thrombophilia patients on antiplatelet therapy with a previous PFO-associated stroke compared to antithrombotic treatment alone if the procedure aligns with the patient’s personal preferences. PFO closure is not suggested in cases without a previous PFO-associated stroke [20]. Such discrepancies in the management of patients with thrombophilia and PFO-related strokes are well depicted in physicians-based surveys [103].

## 6. Conclusions

Transcatheter PFO closure in older patients, patients with thrombophilia, migraines, or DCI has shown promising results in small studies. However, due to the lack of robust data from large RCTs, recommendations remain off-label and unclear. Therefore, there is a pressing need for prospective observational studies and RCTs to identify high-risk phenotypes and subpopulations that might benefit more from closure. Given the current limitations in the literature to provide definite quantitative answers, the decision regarding PFO closure should be approached with an individualised and multidisciplinary shared decision-making framework. This approach enables a thorough assessment of the risk-benefit ratio and feasibility on a case-by-case basis, ensuring careful consideration of each patient’s unique characteristics.

## Figures and Tables

**Table 1:** Selected studies comparing intervention and conservative management for DCS prevention.

Authors	Year	Number of Patients with PFO: Closure/Control	Study Design	Prevention Type	Mean Follow-Up (Years)	Outcome
Billinger et al. [34]	2011	26/39	Prospective	Secondary	5.3	Closure prevents symptomatic and asymptomatic events
Honěk et al. [35]	2014	20/27	Not specified	Primary	None	No difference in venous bubble occurrence but arterial emboli elimination after simulated dives
Henzel et al. [36]	2018	11/0	Retrospective	Secondary	7.6	PFO closure was reasonably effective in the secondary prevention of DCI
Koopsen et al. [37]	2018	21/14	Retrospective	Secondary	6.8	PFO closure was effective and safe for secondary prevention with a safe return to unrestricted diving
Anderson et al. [38]	2019	42/23	Prospective	Primary *n* = 11/ Secondary *n* = 54	6	Healthy divers with large PFOs and relatively serious DCS who will practice advanced diving may benefit more from closure
Vanden Eede et al. [39]	2019	59/0	Retrospective	Secondary	10	Divers undergoing PFO are not completely protected, with 7% recurrence after closure
Honěk et al. [40]	2020	55/98	Prospective	Secondary	7.1	Closure in high-grade PFO was more effective than the conservative approach

DCS = decompression sickness, PFO = patent foramen ovale, DCI = decompression Illness.

**Table 2:** Selected studies assessing PFO closure for migraine patients.

Authors	Year	Number of Patients with PFO: Closure (MA+, MA-)/Control (MA+, MA-)	Study Design	Mean Follow-Up (m)	Postprocedural Treatment	Outcome
Anzola et al. [63]	2006	50 (33, 17)/27 (21, 6)	Prospective	12	Aspirin 300 mg/d × 6 m	Closure significantly improves overall migraine occurrence compared to medication
Vigna et al. [64]	2009	53 (23, 30)/29 (13, 16)	Prospective	16	Clopidogrel 75 mg/d × 3 m + Aspirin 100 mg/d × 6 m	Significant decrease in migraine frequency and severity
Rigatelli et al. [65]	2010	40 (32, 8)/46 (10, 36)	Prospective	29.2	None	Very significant reduction in migraine after PFO closure
Biasco et al. [66]	2014	89 (67, 22)/128 (82, 46)	Retrospective	46.6	Clopidogrel 75 mg/d × 3 m + Aspirin 100 mg/d × 6 m	No difference in migraine evolution, migraine elimination was higher in the closure group
Xing et al. [67]	2016	125 (34, 91)/116 (25, 91)	Prospective	12	Aspirin 100 mg/d × 6 m	PFO closure was more effective compared to medical treatment based on the Headache Impact Test-6
He et al. [68]	2019	91 (91, 0)/101 (101, 0)	Retrospective	60	none	PFO closure was effective in alleviating migraine disability
Trabattoni et al. [57]	2022	62 (62, 0)/0	Prospective	12	Aspirin 100 mg/d × 6 m	Platelet activation that could play a role in MA+ symptoms is switched off after PFO closure
Tong et al. [46]	2023	39 (0, 39)/-	Cross-sectional	20	Antiplatelet (not specified)	Closure significantly improved migraine symptomatology

MA+ = migraine with aura, MA- = migraine without aura, m = months, d = day.

**Table 3:** Selected studies comparing PFO closure vs. medical treatment after cryptogenic stroke in patients <60 and ≥60 years old.

Authors	Year	Number of Patients: ≥60/55 Years Old (Closure/No Closure), <60/55 Years Old (Closure/No Closure)	Study Design	Follow-Up (Years)	Postprocedural Treatment	Outcome
Scacciatella et al. [77]	2016	151 (151/0), 307 (307/0)	Prospective	4.5 (mean)	Dual antiplatelet regimen × 3 m followed by single antiplatelet regimen for ≥3 m	PFO closure was as safe as in younger (>55 years old) patients, recurrent cerebral ischemia after closure was more frequent in older patients
Takafuji et al. [78]	2019	14 (14/0), -	Retrospective	2.6 (mean)	Prescribed in an individual manner	PFO closure was as effective as in younger patients
Wintzer et al. [79]	2020	90 (90/0), 385 (385/0)	Prospective	8 (median)	Aspirin (indefinitely) ± clopidogrel × 6 m	PFO closure was safe and associated with a low rate of recurrence
Kwon et al. [76]	2021	34 (13/21), 86 (47/39)	RCT	<60 + PFO closure: 2.5, <60 + no closure: 2.9, ≥60 + closure: 4.4, ≥60 + no closure: 2.5 (median)	aspirin 100 mg/d + clopidogrel 75 mg/d for ≥6 m (reccomended regimen)	PFO closure was more beneficial in patients ≥60 years old compared to younger ones
Poli et al. [80]	2021	71 (43/28), 123 (103/20)	Prospective	2.8 (mean)	Aspirin + clopidogrel, possible de-escalation to single regimen after 3 or 6 m	Similar recurrence incidence between the groups
Nachoski et al. [81]	2021	101 (101/0), 192 (192/0)	Retrospective	3.6 (mean)	Aspirin 100 mg/d + clopidogrel 75 mg/d × 3 m followed by aspirin monotherapy for ≤6 m	No differences in safety and effectiveness between the groups
Alperi et al. [82]	2022	388 (388/0), 883 (883,0)	Retrospective	3 (median)	Prescribed in an individual manner	PFO closure was safe, with a low but higher stroke recurrence rate compared to younger patients
Chen et al. [83]	2023	78 (35/43), 95 (62/33)	Prospective	2.5 (mean)	According to clinical guidelines	Reduced risk for the primary outcome after closure in the total cohort; elderly patients with closure had a better functional outcome at 180 days compared to controls

**Table 4:** Selected studies comparing PFO closure vs. medical treatment after cryptogenic stroke in patients with and without thrombophilia.

Authors	Year	Number of Patients: PFO Closure (with/without Thrombophilia), Control (with/without Thrombophilia)	Thrombophilia Type	Prevention (Primary/ Secondary)	Study Design	Follow-Up (m)	Postprocedural Treatment for Thrombophilia Patients	Outcome
Giardini et al. [98]	2004	72 (20/52), 0	NA	Secondary	Retrospective	19 (median)	Warfarin × 6 m	PFO closure is effective in recurrence prevention
Kefer et al. [99]	2012	175 (28/147), 0	HHcy 57.1%, PTM 7.1%, protein C deficiency 3.6%, protein S deficiency 17.9%, FVL 10.7%, aCL antibodies 3.6%, aPL 3.6%	Secondary	Prospective	60 (mean)	LMWH × 4–6 weeks + aspirin for 6m	PFO closure is safe and effective even in thrombophilia
Liu et al. [94]	2020	383 (89/294), 208 (45/163)	NA	Secondary	Prospective	53 (median)	Warfarin × 3 m for single embolic event, lifelong warfarin for ≥2 embolic events	PFO closure reduced the risk of event recurrence compared to medical treatment alone
Buber et al. [100]	2021	136 (85/51), 0	APS 31%, FVL 22%, PTM 18%, protein S deficiency 7%, MTHFR mutation 5%, ET 2%	Primary	Retrospective	46 (mean)	Individual antithrombotic therapy maintenance	PFO closure was associated with a significantly lower risk of stroke/TIA
Ben-Assa et al. [101]	2021	800 (239/561), 0	APS 34.3%, Lp(a) elevation 32.6%, HHcy 9.6%, Protein S deficiency 12.6%, FVL 1.3%, PTM 9.2%, AT-III deficiency 7.1%, Protein C deficiency 2.9%	Secondary	Retrospective	41.9 (median)	Warfarin × 3 m followed by lifelong aspirin 100mg/d for 1 thrombotic episode and non-arterial hypercoagulable state; otherwise, lifelong warfarin	PFO closure was safe and effective in thrombophilia patients
Abrahamyan et al. [102]	2023	669 (174/495)	Protein C deficiency 27%, Protein S deficiency 37.9%, AT-III deficiency 20.1%, FVL 19%, PTM 12.1%, aCL antibodies 9%, LAC 5%	Secondary	Retrospective	139.2 (median)	Dual antiplatelet therapy × 6 m followed by aspirin (suggested)	No difference in long-term adverse events between the groups

HHcy = hyper-omocysteinemia, PTM = prothrombin gene mutation, FVL = Factor V Leiden, aCL = anticardiolipin, aPL = antiphospholipid antibodies, LMWH = low-molecular-weight heparin, MTHFR = methylenetetrahydrofolate, ET = essential thrombocytosis, LP = lipoprotein, AT = antithrombin, LAC = Lupus anticoagulant.

## Data Availability

No new data were created or analyzed in this study. Data sharing is not applicable to this article.
